# Cutting through the Clutter: How the Brain Learns to See

**DOI:** 10.1371/journal.pbio.0030256

**Published:** 2005-06-07

Most of us don't have much trouble recognizing what we see. Whether it is a face in a crowd, a bird in a tree, or papers on a desk, our brains expertly distinguish the target from the clutter. It is a simple skill most of us take for granted, but object recognition is not hard-wired. As we navigate our environment, the brain's visual centers continually reorganize themselves, classify novel features, and learn to pick out important objects from the background. Just how the human brain does this is not well understood, but new research by Zoe Kourtzi and colleagues may have uncovered some important clues.

To investigate how the human brain learns to separate targets (signal) from noise Kourtzi et al. showed subjects pictures of novel shapes embedded in a cluttered background and asked the subjects to determine whether or not the shapes were symmetrical. The researchers recorded the subjects' responses while using functional magnetic resonance imaging (fMRI) to measure neuronal activity in brain regions associated with visual processing. Each subject was tested using two sets of novel shapes: high-salience shapes (shapes easily distinguished from the background), and low-salience shapes (shapes camouflaged by the background). After the initial testing, the subjects were trained to recognize a subset of the new shapes from each group, and then re-tested.

Visual input is thought to go through a hierarchy of processing centers that transform retinal images into complex objects and scenes. Kourtzi et al. recorded responses from both early (V1, V2, Vp, and V4) and late (lateral occipital cortex) stages of visual analysis in 26 subjects. The authors found that subjects demonstrated an increased number of correct responses for shapes they encountered during the training sessions, regardless of the type of background the shapes were presented on. By contrast, the fMRI responses differed dramatically, depending on whether the surroundings made the shapes easy or difficult to detect. Low-salience shapes triggered an increased fMRI response across all brain regions following training; high-salience shapes precipitated a decrease in fMRI response in the regions of the lateral occipital cortex, but produced no change in any of the early visual areas (V1, V2, Vp, and V4). [Fig pbio-0030256-g001]

**Figure pbio-0030256-g001:**
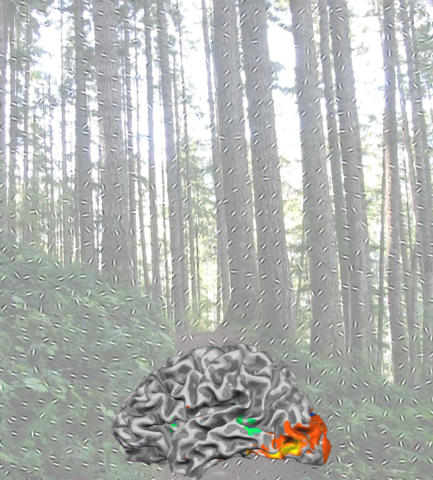
The human brain learns to detect the contours of target objects in cluttered scenes by recruiting early and higher centers of visual analysis

These results demonstrate that the ability to learn to detect novel shapes is independent of the degree of difficulty, but suggest that the brain employs different mechanisms of perceptual learning depending on whether the objects stand out from their surroundings, or are obscured by them. Learning to detect highly camouflaged shapes results in increased brain activity levels that are presumed to reflect an increase in signal processing at the level of both the early visual areas and higher levels of cortical analysis. On the other hand, the reduction of neural activity that occurs during learning of more distinctive shapes likely reflects efficient neural coding of the critical features for their recognition at later stages of visual analysis.

According to Kourtzi and her colleagues, their results provide evidence that the visual brain is capable of tailoring the mechanism of perception to best suit the task. When the signal is weak—as in the case of viewing camouflaged targets—learning amplifies neural responses to the target shapes and drowns out the noise. But when the signal is strong—as in the case of viewing easily distinguishable, highly salient targets—neural activity in the visual cortex is reduced, possibly because training engages smaller populations of neurons that respond much more selectively to distinctive features of the stimulus.

In other words, all visual stimuli are not treated equally, and with just cause: the brain's unique ability to treat ambiguous signals differently than robust ones likely allows it to optimize neural coding, and in doing so, learn to increase detection of a broad spectrum of visual signals.

